# Factors Associated with the Unplanned Pregnancy at Suhul General Hospital, Northern Ethiopia, 2018

**DOI:** 10.1155/2020/2926097

**Published:** 2020-06-27

**Authors:** Yohannes Moges, Solomon Adanew Worku, Abrhaley Niguse, Bayew Kelkay

**Affiliations:** ^1^Department of Midwifery, Institute of Medicine and Health Science, Debre Berhan University, Debre Birhan, Ethiopia; ^2^Department of Midwifery, College of Health Science and Comprehensive Specialized Hospital, Aksum University, Axum, Ethiopia; ^3^School of Midwifery, College of Medicine and Health Science, University of Gondar, Gondar, Ethiopia

## Abstract

**Background:**

Unplanned pregnancy is a fundamental concept that is used to recognize the fertility of populations and the unmet need for contraception and family planning. Unplanned pregnancy happened mainly due to the results of not using contraception or inconsistent or incorrect use of effective methods. Reducing the number of unplanned pregnancy endorses reproductive health mainly by reducing the number of times a woman is exposed to the risk of pregnancy and childbearing.

**Objective:**

This study is aimed at assessing the magnitude of unplanned pregnancy and associated factors among pregnant mothers attending antenatal care at Suhul General Hospital, Northern Ethiopia.

**Methods:**

A facility-based cross-sectional study design was conducted among pregnant mothers visiting antenatal care follow-up from February to April 2018 at Suhul General Hospital, Shire, and Northern Ethiopia. The study participants were selected using a systematic sampling method, and the data was collected using a pretested structured questionnaire through face-to-face interviews. Bivariate and multivariate logistic regression analyses were done to determine the association of each independent variable with the dependent variable.

**Result:**

The magnitude of unplanned pregnancy among 379 pregnant mothers was 20.6%. Unmarried women [AOR: 4.73, 95% CI: (1.56, 14.33)], age above forty [AOR: 4.17, 95% CI: (1.18, 14.6)], had no history of unplanned pregnancy [AOR: 3.26 95% CI: (1.65, 6.44)], and unemployed [AOR: 6.79; 95% CI: (2.05, 22.46)] were the variables significantly associated with the magnitude of unplanned pregnancy. *Conclusion and Recommendation.* The findings of this study showed that the magnitude of unplanned pregnancy was high and age, marital status, occupation, and history of unplanned pregnancy were statistically associated with an unplanned pregnancy. There is seeming necessity to plan strategies of communication within couples or individuals on reproductive especially on fertility and promote family planning methods.

## 1. Introduction

An unplanned pregnancy is a pregnancy that is either unwanted (that is, the pregnancy occurred when no children or no more children were desired) or the pregnancy is mistimed (that is, the pregnancy occurred earlier than desired). Sexual activity without the use of contraception through choice or coercion is the predominant cause of unplanned pregnancy. Unplanned pregnancy is a worldwide problem with significant adverse consequences for women, their families, and society. In developing country settings, women who seek to abort unintended pregnancy face the risk of serious long-term health effects, including infertility and maternal death [1].

Globally, in 2014 of the total pregnancy, 44% of pregnancies were unplanned, in which, more than half (56%) of unplanned pregnancies ended in abortion [2]. Worldwide, 303,000 mothers died in 2015; of this, 99% occurred in 2015 in developing counties, while sub-Saharan Africa alone accounting for roughly 66%, followed by Southern Asia (22%). Worldwide, around 26.5 million unplanned pregnancies happened each year due to the incorrect use or failure of contraceptives [3].

Annually, six million unplanned pregnancies, 2.1 million unplanned births, 3.2 million abortions, and 5,600 maternal deaths would be reduced by avoiding the unmet need for modern contraception [4]. According to the 2017 World Health Organization report, an average of 56 million pregnancies were terminated worldwide each year, 49.3 million in developing regions and 6.6 million in developed regions, which is 25% of all pregnancies ended in abortions. In developing countries, every year, around 22,000 women lost their lives due to the complication of abortion [5].

In Ethiopia, unplanned pregnancy indicates that it is one of the major reproductive health problems with all its adverse outcomes. Every year, on average, 4.93 million pregnancy happened and 1.9 million (38%) Ethiopian women have an unplanned pregnancy; of which, 620,296 induced abortions were done; 103,648 women were treated for complications of such abortions [6].

The risk factors associated with unplanned pregnancy include unwanted pregnancy in adolescents, inadequate family planning services, low socioeconomic status, and lack of or improper use of family planning methods and unawareness of it. Unplanned pregnancy is associated with an increased risk of problems for the mother and fetus [7]. If a pregnancy is not planned before conception, a woman may not be in optimal health for childbearing. Women with an unplanned pregnancy could delay prenatal care that may affect the health of the baby. In many developing countries, poverty, malnutrition, and lack of sanitation and education contribute to serious health consequences for women and their families experiencing an unplanned pregnancy. The concept of unplanned pregnancy helps in understanding the fertility of populations and the unmet need for contraception [8].

## 2. Materials and Methods

### 2.1. Study Design and Population

Facility-based cross-sectional study design was conducted from February to April 2018 with 379 mothers who are attending antenatal care at Suhul General Hospital, Northern Ethiopia. The study was conducted at Shire town, the capital of northwest zone of Tigray regional state located 1087 Kms far from the capital city of Ethiopia, Addis Ababa, and 357 Kms from Mekelle, the capital of regional. Based on the reports from the zonal administrative office, the town has 67,243 total populations; among these, 36,146 are females. In Suhul General Hospital, the total women who attend antenatal care in 2017/18 were 5,148 and the recent quarter report 1278.

### 2.2. Inclusion and Exclusion Criteria

All pregnant women who came for antenatal care service during the study period were included in the study.

### 2.3. Sample Size and Sampling Technique

The sample size was determined by using the single-population proportion formula with the following assumptions: 5% level of significance (*a* = 0.05), 5% margin of error (*d* = 0.05), P is the prevalence of unplanned pregnancy is equal to 33.7% taken from a study conducted in Hawassa city [9] and 10% nonresponsive rate were taken into account. Based on this, the total sample of the study was 379. Finally, the pregnant women were selected by a systematic random sampling technique. The skip interval was calculated for selecting interviewed participants systematically as *K* = *N*/*n*, where *K* is the skipping interval and it was 3. The starting point was selected randomly and then beginning from the starting point, and every third woman at the exit of the health facility was included.

### 2.4. Study Variables

Unplanned pregnancy is the dependent variable. Sociodemographic variables (age, marital status, monthly income, educational level, partner educational level, religion, occupation, and residence) reproductive factors (history of pregnancy, history of abortion, history of unplanned pregnancy, parity, and number of alive children), and family planning-related factors (heard of family planning, decision maker, and history of contraceptive use) are the independent variables.

### 2.5. Data Collection and Quality Control

The data were collected by using a pretested structured questionnaire through the face-to-face interview from the pregnant women after getting written informed consent from pregnant women. Questionnaires were prepared first in English after reviewing different works of literature and translated into the local language (Tigrigna) and then back to English by the third party. The questionnaire was constructed based on the variables to achieve the objectives of the study. Four midwives who were fluent in the local language were involved in data collection. Detail orientation was provided for data collectors.

### 2.6. Data Processing and Analysis

EP info version 7.2 was used for data entry and analyzed using SPSS version 20 software. Descriptive statistics were also carried out for frequencies. Bivariable and multivariable logistic regression analyses were done. Bivariate analysis from analytical statistics was applied, and variables showing *p* value < 0.05 were taken for multivariable logistic regressions. Adjusted odds ratio (AOR) with their 95% confidence intervals was computed to identify the presence of association. Variables at *p* value < 0.05 in multivariable logistic regressions were considered significant association with the outcome.

### 2.7. Data Quality Assurance

The training was provided by the principal investigator to the data collectors and supervisors. The study used the properly designed data collection tool and pre-tested questionnaire. Supervisors and principal investigator checked the completeness and correctness of each questionnaire daily.

## 3. Result

### 3.1. Sociodemographic and Economic Characteristics of the Respondents

A total of 379 pregnant women responded to the questionnaire which makes the response rate of 100%. The majority of the respondents 92(24.3%) were in the age group of 25-29 followed by 30-34, 86 (22.7%). While considering the mean age of the respondents, it was 29. Regarding the marital status of the respondents, the majority of the respondents 302 (79.7%) were married. The religion of most of the respondents was orthodox 268 (70.7%). Concerning the educational level, the majority of respondents were postsecondary school level 124 (32.7%). According to the partner educational level of the respondents, majority of them were postsecondary school levels 149 (49.3%). Regarding the occupation of the respondents, majority of them was government employees 125 (33%). Concerning age at first marriage, 35 (9.2%) were married at the age of <18 years, and the majority of the participants lives in an urban area 286 (75.5%). According to the monthly income of the respondents, majority of them had >3000 birr (>111 dollar) 90 (23.7%) (Table 1).

### 3.2. Specific Information regarding Family Planning Service

Three hundred twenty-eight (86.5%) respondents heard about contraceptive, and 256 (67.7%) of the respondents heard information from health workers, and 286 (78%) of the respondent have ever used any type of contraceptive before becoming pregnant. The majority 126 (44%) of them used injectables followed by implants 104 (36.7%). Regarding the decision making to use a contraceptive method, 188 (65.7%) of the respondents were decided by their husband. Of the total respondents, 286 (75.5%) of them had used contraceptive methods. The most common cause of not using contraceptive was child preference 43 (46.3%) followed by fear of side effects 25 (26.9%) (Table 2).

### 3.3. Reproductive History of Pregnant Women

The study finding revealed that among all respondents, 311 (82.1%) had a history of previous pregnancy. The majority of the respondents 143 (46%) had 1-2 previous pregnancies. Among those who had a history of pregnancy, 82 (21.6%) participants had a history of abortion. Of all the respondents, 180 (58.8%) respondents had given birth to 1-2 and 168 (61.0%) of the respondents had 1-2 alive children during the study period. From all of the respondents, 67 (17.7%) had a previous history of unplanned pregnancy. The majority of respondents (49.1%) think that the number of sufficient children for a lifetime was four children (Table 3).

### 3.4. Magnitude of Unplanned Pregnancy

From the total, 78 study participants (20.6%) had an unplanned pregnancy. Failure of contraceptive 21 (26.9%), lack of contraceptive to use (16.7%), missed time (19.2%), and partner preference and divorced (19.2%). Do not have enough money to take care a baby (9%), still in school (5.2%), and rape were the most common motives mentioned by respondents who had an unplanned pregnancy.

### 3.5. Factors Associated with an Unplanned Pregnancy

Bivariate analysis for each independent variable has been used to assess factors associated with the magnitude of unplanned pregnancy, and age, marital status, educational level, occupation, monthly income, heard contraceptive, history of abortion, number of alive children, and history of unplanned pregnancy statistically associated with unplanned pregnancy. Statistical significance accepted at *p* value < 0.05 (Table 4).

The above variables showing *p* value < 0.05 during bivariate analysis were taken to multivariate logistic regressions. Occupation, marital status, age, and history of an unplanned pregnancy were the variables that showed association in multivariate logistic regression. A significant association in multivariate logistic regression is also declared at *p* value < 0.05 (Table 4).

In multivariate analysis, unmarried women showed 4.7 times more likely to have unplanned pregnancy compared to their counterpart [AOR: 4.73, 95% CI (1.56, 14.33)]. Similarly, women more than forty aged were 4 times [AOR: 4.17, 95% CI (1.18, 14.6)] more likely to have unplanned pregnancy compared to women who are in the age group of 15-19.

Women who had never had a history of previous unplanned pregnancy were 3.2-time [AOR: 3.27, 95% CI (1.65, 6.49)] higher odds of having unplanned pregnancy compared to those who had a history of an unplanned pregnancy. Unemployed women had 6.79 times more likely to have an unplanned pregnancy than their complement [AOR: 6.79, 95% CI (2.05, 22.46)].

## 4. Discussion

The magnitude of unplanned pregnancy in the current study was 20.6% [CI: 16.53-24.67]. This study is consistent with studies conducted in Debre Birhan (23%) and Gondar (20.6%) [10, 11]. However, the finding of the current study is lower than the study conducted in Hawassa (34%), Negele (44%), Sudan (30.2%), Malawi (43%), and USA (45%) [9, 12–15], whereas the finding of this study is higher than the study conducted in Belesa (13.7%) [16] and Bahir Dar (13.7%) [17]. This variance might be on account of the difference in the sociodemographic and cultural status of the study areas and time gap.

The probabilities of unintended pregnancies among women above the age of forty were 4 times [AOR: 4.17, 95% CI (1.18, 14.6)] more likely than those women in the age group of 15–19. This finding is consistent with a study conducted in Belesa woreda [16]. This could be due to women above the age of forty are less likely to desire further pregnancies since they likely have the ideal number of children that they want. Besides, advanced maternal age has been associated with an increased risk of obstetrical complications.

The study finding showed that unmarried women were 4.2 times more likely to have unplanned pregnancy compared to married women [AOR: 4.15, 95% CI (1.315, 13.09)]. The current study is in line with the study conducted in Maichew [18] and Debre Birhan towns [10]. The probable reason could be social discrimination for unmarried women to be pregnant and also to utilize contraceptives to prevent pregnancy. Besides, unmarried women were more likely to engage in sexual activity other than childbearing.

In terms of occupation, unemployed women were 6.4 times more likely to have had unplanned pregnancy compared to civil servants. This finding is in line with the study conducted in South Africa [19]. On the contrary, on the study done in Hawassa, the odds of unplanned pregnancy was higher among others as compared to unemployed by 40 times. The probable reason for the difference might be due to unemployed women may have low socioeconomic status, so with limited economic resources, they might have the fear of economic instability and thus the difficulty to support a family.

The odds of having unplanned pregnancy among women who had no history of unintended pregnancy were more likely than those who had a history. On the contrary, in the study done in Bahir Dar Felege Hiwot referral hospital [17] and Hosanna town [20], women who had previously had unplanned pregnancy do not experienced a more unintended pregnancy. This discrepancy might be due to the women who had a history of unplanned pregnancy might have used contraceptives to avoid the recurrence of unintended pregnancy.

## 5. Conclusion

In this study, nearly a quarter of respondents have experienced unplanned pregnancy. Being unmarried, had no history of unplanned pregnancy, having an age greater than forty, and unemployed were a statistically significant association with the unplanned pregnancy.

## 6. Recommendation

There is a seeming necessity to plan strategies of communication within couples or individuals on reproductivity especially on fertility and promotion of family planning methods. In addition, due attention should be given for improving maternal education and to provide insights into how to access and use contraception, especially for unmarried and unemployed women to reduce unintended pregnancy.

## Figures and Tables

**Table 1:** Sociodemographic characteristics of pregnant women attending ANC at the Shire, Suhul General Hospital, Tigray regional state, Ethiopia, 2018 (*n* = 379).

Variables	Category	Frequency (*n*)	Percent (%)
Age	15-19	35	9.2
	20-24	92	24.3
	25-29	74	19.5
	30-34	86	22.7
	35-39	47	12.4
	>44	45	11.9

Marital status	Unmarried	28	7.4
	Married	302	79.7
	Divorced	32	8.4
	Widowed	17	4.5

Religion	Orthodox	268	70.7
	Muslim	83	21.9
	Protestant	17	4.5
	Catholic	11	2.9

Educational level	Unable to read and write	57	15.0
	Able to read and write	51	13.5
	Elementary (1-8) school level	56	14.8
	Secondary (9-12) school level	91	24.0
	Postsecondary school	124	32.7

Partner education level (*n* = 302)	Unable to read and write	20	6.6
	Able to read and write	27	9
	Elementary (1-8) school level	32	10.6
	Secondary (9-12) school level	74	24.5
	Postsecondary school level	149	49.3

Occupation	Housewife	117	30.9
	Government employ	125	33.0
	Private business	89	23.5
	Farmer	29	7.7
	Other	19	5.0

Month income Level	<500 birr (<18.5 dollar)	93	24.5
	500-1000 birr (18.5-37 dollar)	66	17.4
	1001-2000 birr (37.1-74 dollar)	88	23.2
	2001-3000 birr (74.1-111 dollar)	42	11.1
	>30000 birr (>111 dollar)	90	23.7

*n*: number.

**Table 2:** Information regarding family planning methods among pregnant women attending ANC at the Shire, Suhul General Hospital, Tigray regional state, Ethiopia, 2018 (*n* = 379).

Variables	Categories	Frequency (*n*)	Percent (%)
Source of information (*n* = 328)	Health worker	256	78.0
	Relatives	23	7.0
	Mass media	31	9.5
	Written materials	14	4.3
	Others	4	1.2

Types of FP (*n* = 286)	Pills	45	15.7
	Injectable	126	44.0
	Implant	104	36.7
	IUCD	10	3.5
	Others	1	0.3

Decision maker (*n* = 286)	Your self	82	28.7
	Husband	13	4.5
	Both	188	65.7
	Health worker	3	1.0

Reason for why not used FP (*n* = 93)	Lack of information	10	10.7
	Child preference	43	46.3
	Religion view	11	11.9
	Fear of side effect	25	26.9
	Husband domination	2	2.1
	Inaccessibility of service	2	2.1

*n*: number; FP: family planning; IUCD: intrauterine contraceptive device.

**Table 3:** Reproductive history-related among pregnant women attending ANC at the Shire, Suhul General Hospital, Tigray regional state, Ethiopia, 2018 (*n* = 379).

Variables	Categories	Frequency (*n*)	Percent (%)
Number of pregnancy (*n* = 311)	1-2	143	46
	3-4	120	38.6
	5 and above	48	15.4

Number of gave birth (*n* = 306)	1-2	180	58.8
	3-4	100	32.7
	5 and above	26	8.5

Number of alive children (*n* = 275)	1-2	168	61.0
	3-4	91	33.0
	5 and above	16	6.0

Number of sufficient child during a lifetime	One	2	0.5
	Two	18	4.7
	Three	66	17.4
	Four	186	49.1
	Five	59	15.6
	Six more	48	12.7

*n*: number.

**Table 4:** Factors associated with unplanned pregnancy among women attending ANC at the Shire, Suhul General Hospital, Tigray regional state, Ethiopia, 2018 (*n* = 379).

Variables	Category	Unplanned pregnancy frequency (percentage)		COR (95% CI)	AOR (95% CI)
		Yes	No		
Age	15-19	10 (2.64)	25 (6.6)	1	1
	20-24	16 (4.22)	76 (20.05)	1.90 (0.76, 4.72)	2.56 (0.88, 7.48)
	25-29	15 (3.95)	59 (15.6)	1.57 (0.62, 3.98)	1.71 (0.6, 4.89)
	30-34	10 (2.64)	76 (20.05)	3.04 (1.13, 8.15)	3.05 (1.03, 9.02)
	35-39	20 (5.3)	27 (7.1)	0.54 (0.21, 1.37)	0.906 (0.29, 2.74)
	≥40	7 (1.85)	38 (10)	2.17 (1.02, 6.45)	4.17 (1.18, 14.6)^∗^

Marital status	Married	8 (2.1)	9 (2.4)	1	1
	Widowed	9 (2.4)	19 (5)	1.87 (0.543, 6.48)	1.97 (0.48, 8.04)
	Unmarried	42 (11.1)	260 (68.6)	5.503 (2.011, 15.057)	4.73 (1.56, 14.33)^∗^
	Divorced	19 (5)	13 (3.4)	0.608 (0.186, 1.990)	0.58 (0.15, 2.14)

Occupation	Civil servant	9 (2.4)	10 (2.6)	1	1
	Housewife	22 (5.8)	95 (25)	3.886 (1.41, 10.702)	5.4 (1.68, 17.34)
	Unemployed	14 (3.7)	111 (29.3)	7.136 (2.476, 20.561)	6.79 (2.05, 22.46)^∗^
	Private business	24 (6.3)	65 (17.2)	2.438 (0.883, 6.72)	2.87 (0.88, 9.33)
	Farmer	9 (2.4)	20 (5.3)	2.0 (0.605, 6.612)	3.13 (0.74, 13.19)

History of unplanned pregnancy	Yes	26 (6.9)	41 (10.8)	1	1
	No	52 (13.7)	260 (68.6)	3.17 (1.78, 5.63)	3.26 (1.65, 6.44)^∗^

^∗^ *p* value < 0.05, COR: crude odds ratio; AOR: adjusted odds ratio; CI: confidence interval.

## Data Availability

All data generated/analyzed during this study are included in this published article. Besides, part of the row datasets will be available from the corresponding author on a reasonable request.

## Abbreviations

ANC: Antenatal care
AOR: Adjusted odds ratio.
